# Spread of resistant gram negatives in a Sri Lankan intensive care unit

**DOI:** 10.1186/s12879-017-2590-7

**Published:** 2017-07-11

**Authors:** Kavinda Tissera, Veranja Liyanapathirana, Nilanthi Dissanayake, Vasanthi Pinto, Asela Ekanayake, Manjula Tennakoon, Dinuka Adasooriya, Dulmini Nanayakkara

**Affiliations:** 1 Postgraduate Institute of Science, University of Peradeniya, Peradeniya, Sri Lanka; 2 Department of Microbiology, Faculty of Medicine, University of Peradeniya, Peradeniya, Sri Lanka; 3 Department of Anaesthesiology and Critical Care, Faculty of Medicine, University of Peradeniya, Peradeniya, Sri Lanka; 4 Faculty of Allied Health Sciences, University of Peradeniya, Peradeniya, Sri Lanka

**Keywords:** Multi drug resistance, RAPD, Sri Lanka

## Abstract

**Background:**

Infections with multi drug resistant (MDR) organisms are a major problem in intensive care units (ICUs). Proper infection control procedures are mandatory to combat the spread of resistant organisms within ICUs. Well stablished surveillance programmes will enhance the adherence of the staff to infection control protocols. The study was conducted to assess the feasibility of using basic molecular typing methods and routine hospital data for laboratory surveillance of resistance organisms in resource limited settings.

**Methods:**

A retrospective study was conducted using consecutive Gram negative isolates obtained from an ICU over a six month period. Antibiotic sensitivity patterns and random amplified polymorphic DNA (RAPD) based typing was performed on the given isolates.

**Results:**

Of the seventy isolates included in the study, seven were *E.coli*. All *E.coli* were MDRs and Extended Spectrum β lactamse (ESBL) producers carrying *bla*_CTX-M_. Fourteen isolates were *K.pneumoniae*, and all were MDRs and ESBL producers. All *K.pneumoniae* harboured *bla*_SHV_ while 13 harboured *bla*_CTX-M_. The MDR rate among *P.aeruginosa* was 13% (n=15) while all acinetobacters (n=30) were MDRs. Predominant clusters were identified within all four types of Gram negatives using RAPD and the ICU stay of patients overlapped temporally.

**Conclusion:**

We propose that simple surveillance methods like RAPD based typing and basic hospital data can be used to convince hospital staff to adhere to infection control protocols more effectively, in low and middle income countries.

## Background

In many developing countries, improper use of antibiotics, poor sanitation and shortfalls in infection control facilitate the amplification and dissemination of resistant strains in health care institutes and the community [1]. Increasing air travel, global mobility, health tourism, animal exports and international trade facilitate the spread of resistant organisms to countries worldwide [2]. Therefore, antimicrobial resistance is a global crisis that no single country can fight against, in isolation.

The World Health Organization (WHO) in February 2017 published a list of priority organisms that needs new antibiotics urgently. These include carbapenem resistant *Acinetobacter baumanii*, carbapenem resistant *Pseudomonas aeruginosa* and enterobacteriaceae resistant to carbapenems and 3^rd^ generation cephalosporins at critical level, emphasizing the magnitude of the problems occurring due to these [3]. Gram-negative bacteria, including the above were also the most common cause of healthcare associated infections (HAIs) in many low and middle income countries, and the focus of this paper [4].

Antibiotic resistance is a major concern in the Sri Lankan health care sector, particularly in relation to HAIs, with higher rates of cephalosporin resistance than community isolates [5]. Carbapenem resistance is also known to be widespread among many institutions [6–8].

Intensive care units (ICUs) have a high antibiotic pressure. Therefore, resistant organisms develop and thrive in these environments. High antibiotic resistance in ICU settings is present throughout Sri Lanka [7–10]. While most studies describe the sensitivity patterns, mechanisms of resistance, genetic determinants of resistance and clonality have not been studied widely in Sri Lanka. Currently, surveillance also remains at the level of identification of organisms and resistance patterns via routine testing. Surveillance is considered as an integral part of infection prevention and control programmes [11]. Effective surveillance programmes are known to reduce rates of hospital acquired infections [12]. Despite the recent improvement of laboratory infra-structure in Sri Lanka, no routine typing protocols are in place. While methods such as Next Generation Sequencing (NGS) based typing and Multi Locus Sequence typing (MLST) have now become the standard methods in typing, in resource limited settings, simpler methods such as random amplification of polymorphic DNA (RAPD) can be used more cost-effectively.

This study was carried out in order to identify the feasibility of using RAPD and minimal clinical data for laboratory surveillance in resource limited settings.

## Methods

This laboratory based study was conducted on consecutive Gram negative isolates obtained from cultured respiratory tract specimens received from patients admitted to the ICU of Teaching Hospital Peradeniya (THP) from March to September 2015. The study unit is the single ICU providing intensive care facilities to this 924 bed hospital and has 10 ICU beds that are housed in three compartments. The first isolate obtained per species was selected per patient unless there was a gap of >5 days between the two specimens or a difference in the morphological appearance or antibiogram. Ethical clearance for the study was obtained from the Institutional Ethical Review Committee, Faculty of Medicine, University of Peradeniya, Sri Lanka.

Isolates were stored at −80 °C prior to testing. Date of admission to the ICU and date of discharge were obtained retrospectively from the institutional records.

All isolates were identified up to their species level except the *Acinetobacter* spp., by a using series of standard biochemical tests supplemented by commercial kits where needed. *Acinetobacter* spp. were identified up to genus level.

Antibiotic susceptibility testing (ABST) was performed and interpreted accordingly to the Clinical and Laboratory Standards Institute (CLSI) guidelines and standards (CLSI, 2016). ESBL production was confirmed in *E. coli* and *K. pneumoniae* isolates by the combined disc test [13]. Carbapenem non-susceptible (either resistant or intermediate sensitive) *E.coli* and *K. pneumoniae* isolates were tested for carbpenemase production by the modified Hodge test (MHT).

As genetic determinants of ESBL production, *bla*_CTX-M_, *bla*_SHV_, and *bla*_TEM_ genes were detected using previously described primers in all *E. coli* and *K. pneumoniae* isolates [14]. PCR reactions were carried out with 2 μL of template obtained from boil lysis of organisms, 5 μL of 5× Green GoTaq™ buffer (Promega, USA), 3 μL of 25 mM MgCl_2_ (Promega, USA)_,_ 1 μL of 10 mM dNTP mix (Promega, USA), 0.4 μL of 5 U/μL GoTaq™ Flexi DNA polymerase (Promega, USA) and 0.5 μL of each 10 μM forward and reverse primers (Integrated DNA Technologies) with molecular grade water (Invitrogen, USA) added to a total of 25 μL. The annealing temperature for the ESBL multiplex PCR was 60 °C.

Six primers were screened using DNA extracted from *K. pneumoniae* BAA 1705, *E. coli* ATCC 25922, *Pseudomonas aeruginosa* ATCC 27853 and a clinical *Acinetobacter* spp. isolate (isolate no 105). The primer giving rise to the highest number of discrete bands was selected for typing the given group of organisms. Selected primers were RAPD_1:GAAGCAGCCCGGTAGTAGGTTGAG for *E. coli* and *K. pneumoniae* [15], RAPD_5: AGCGGGCCAA for *Acinetobacter* species and RAPD_6: ACGGCCGACC for *Pseudomonas aeruginosa* [16]. The PCR mater-mix constituted of 3 μl of 25 nM MgCl_2_ (Promega, USA), 5 μl of 5× Green GoTaq Buffer (Promega, USA), 1 μl of 10 mM dNTP mix (Promega, USA), 0.3 μl 5u/ μl of GoTaq Flexi DNA polymerase, 2 μl of 10 mM primer (Integrated DNA Technologies), 2 μl of template DNA and 11.7 μl of molecular grade water (Invitrogen, USA) to a total volume of 25 μl. All reactions were performed at an annealing temperature of 36 °C for 1 min.

All gel pictures captured were analyzed with the GelJ software downloaded from http://sourceforge.net/projects/gelj/ on 30/01/2016 [17]. Dendograms were drawn with Dice co-efficient for similarity calculations using UPGMA linkage. Tolerance levels were adjusted for the given species of isolates with the reference lanes.

## Results

The mean stay until samples collection, in instances where data could be retrieved (*n* = 56), was 7.4 days (SD 6.8), while the median was 5 days (range 0 to 33 days). The mean length of ICU stay was 13.4 days (SD 10.1), while the median was 9 days (Interquartile range 3–9.75 days). Fifteen patients were transferred back to ward (28.8%) while 37 patients (71%) died during the ICU stay (data available in 52 patients).

During the six month study period a total number of 70 Gram negative isolates were obtained from respiratory tract specimens. Of these 70 isolates, seven isolates were *E. coli* (10%), 14 were *K. pneumoniae* (20%), three isolates were *Stenotrophomonas maltophilia* (4.3%) and one was *Aeromonas hydrophila* (1.4%). Also 15 of the 70 isolates were identified as *P. aeruginosa* (21.4%) *and* 30 were identified as *Acinetobacter* spp. (42.9%).

### *E. coli* isolates

All *E.coli* isolates were resistant to ampicillin, cefotaxime, ceftriaxone and aztreonam. Six isolates (85.7%) were resistant to ceftazidime and cefipime. Two (28.6%) isolates were resistant to piperacillin-tazobactam. One isolate (14.3%) was resistant to both imipenem and meropenem while one isolate (14.3%) was resistant to meropenem only. Six isolates (85.7%) were resistant to ciprofloxacin while five isolates (71.4%) were resistant to levofloxacin. All isolates were sensitive to amikacin while two (28.6%) were resistant to gentamicin. Using the commonly used definition of multi drug resistance (MDR) as an organism being resistant to three or more classes of antibiotics [18], all seven *E.coli* isolates were identified as MDRs. All seven isolates were confirmed to be ESBL producers by the combined disc test while one isolate (14.3%) was identified as a carbapenamase producer by the MHT. All seven isolates were found to harbour *bla*_CTX-M_ while one isolate each harboured *bla*_SHV_, and *bla*_TEM_ as well (Table 1).

**Table 1:** Positivity rates for genetic determinants of ESBL production

Isolate type	*bla*_CTX-M_	*bla*_SHV_	*bla*_TEM_
*E. coli* (*n* = 7)	100% (*n* = 7)	14.3% (*n* = 1)	14.3% (*n* = 1)
*K. pneumoniae* (*n* = 14)	92.9% (*n* = 13)	100% (*n* = 14)	57.1% (*n* = 8)

**Fig. 1:**
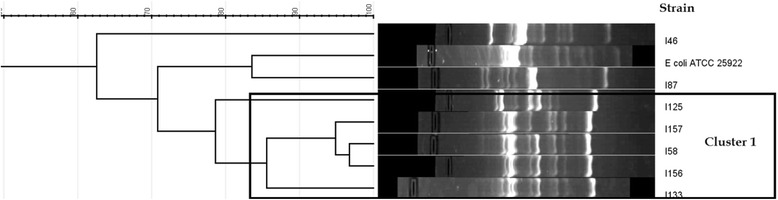
RAPD cluster analysis of the *Escherichia coli* isolates

The UPMGA based clustering of the RAPD results of the *E. coli* isolates (*n* = 7) with a tolerance of 4.5 at a 75% similarity revealed a single cluster consisting of 5 (71.4% of the *E.coli* isolates) isolates while the remaining two isolates were singletons (Fig. 1). As the number of isolates were small, no further analysis was performed.

**Fig. 2:**
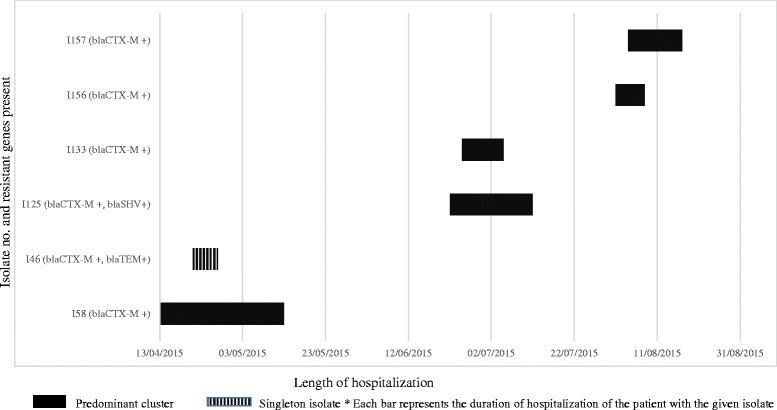
Timeline of ICU stay in patients with *E.coli* isolates

Analysis of overlap of stay up to the date of discharge/death in the six instances where data was available identified that in three occasions, there was on epidemiological link i.e. an overlap in hospital stay between two patients (Fig. 2).

### *K. pneumoniae* isolates

All *K. pneumoniae* tested were resistant to cefotaxime, ceftriaxone, ceftazidime and aztreonam. Eleven isolates (78.6%) were resistant to cefepime and piperacillin-tazobatam. Five isolates (35.7%) were resistant to imipenem, meropenem or both while one was intermediate sensitive. Ten isolates (71.4%) were resistant to gentamicin while one isolate (7.1%) was resistant to amikacin. Twelve (85.7%) were resistant to ciprofloxacin while eight (57.1%) were resistant to levofloxacin. All *K.pneumoniae* isolates were MDRs. All *K. pneumoniae* isolates were confirmed to be ESBL producer while six *K. pneumoniae* isolates (42.8%) that were non-susceptible for carbapenems were identified as carbapenamase producers by the MHT. All isolates were positive for *bla*_SHV_ gene while 13 (92.8%) were found to carry *bla*_CTX-M_ gene and eight were found to carry *bla*_TEM_ (Table 1). Seven isolates (50%) harboured all three determinants.

**Fig. 3:**
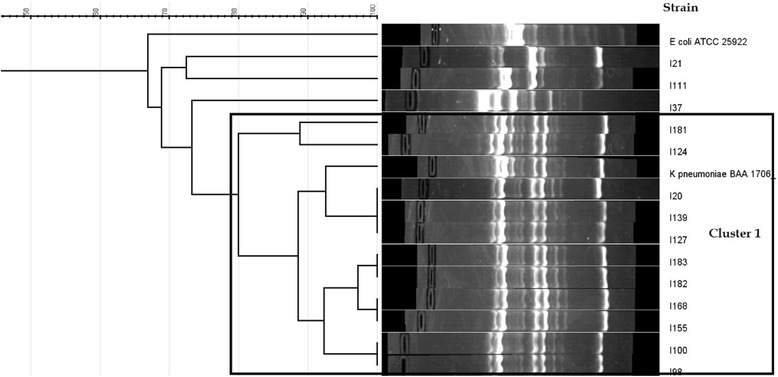
RAPD cluster analysis of the *Klebsiella pneumoniae* isolates

The UPMGA based clustering of the RAPD results of the *K. pneumoniae* isolates (*n* = 14) with a tolerance of 4.5 at a 80% similarity revealed a single cluster consisting of 11 (78.6% of the *K. pneumoniae* isolates) isolates while the rest of the isolates were grouped in twos or as singletons (Fig. 3).

**Fig. 4:**
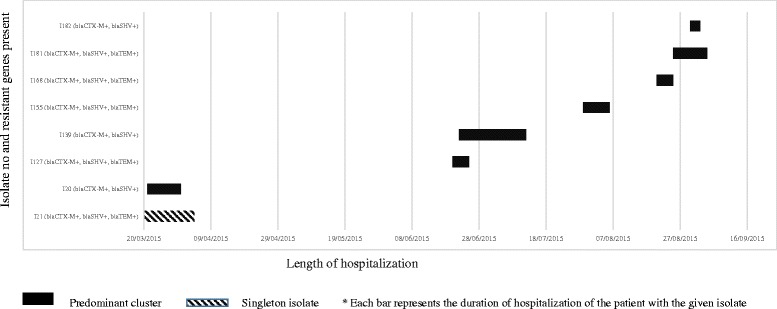
Timeline of ICU stay in patients with *K.pneumoniae* isolates

Data on discharge was available in eight patients. There were overlaps in stay in three occasions, including one where the organisms belonged to different RAPD clusters, while in one instance the occurrence was in isolation (Fig. 4).

### *P. aeruginosa* isolates

Out of 15 *P. aeruginosa* tested no resistant isolates were found for ceftazidime and cefepime. One isolate (6.7%) was resistant to aztreonam. Two isolates (13.3%) were found to be resistant to imipenem, meropenem and piperacillin-tazobactam. One isolate (6.7%) was found to be resistant to gentamicin while no resistance was detected against amikacin. Only two isolates (13.3%) were found to be MDR.

**Fig. 5:**
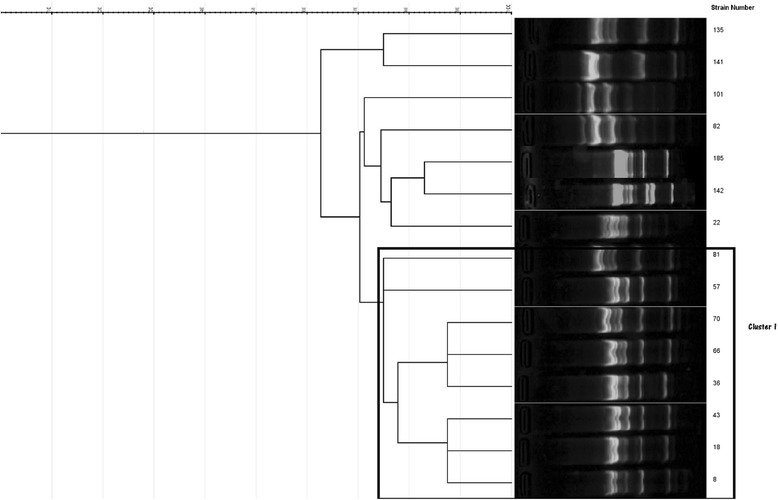
RAPD clusters of the *Pseudomonas aeruginosa* isolates

The UPMGA based clustering of the RAPD results of the *P. aeruginosa* isolates (*n* = 15) with a tolerance of 4 at a 75% similarity revealed a single cluster consisting of 8 (53.3% of the *P. aeruginosa* isolates) isolates while the rest of the isolates grouped in twos or as singletons (Fig. 5).

**Fig. 6:**
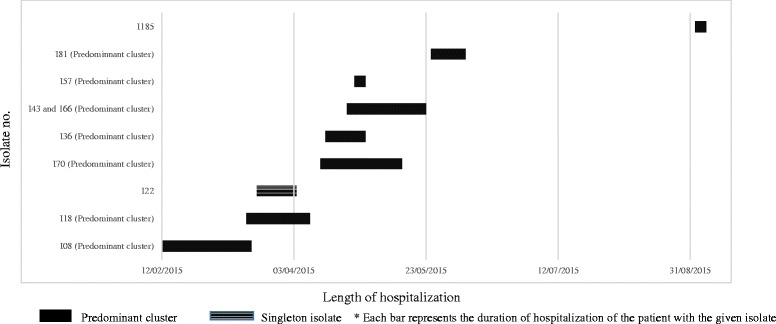
Timeline of ICU stay in patients with *P.aeruginosa* isolates

Many of the pseudomonas isolates of the predominant cluster occurred during an overlapping time period (Fig. 6).

### *Acinetobacter* isolates

All *Acinetobacter* spp. were MDRs and resistant to cefotaxime, imipenem, meropenem, and piperacillin-tazobactam. Twenty eight isolates (93.3%) were resistance to ceftazidime while 29 (96.7%) isolates were resistant to cefipime. All isolates were resistant to ciprofloxacin. Gentamicin resistance was present among 26 isolates (86.7%) while amikacin resistance was present in 16 isolates (53.3%). Twenty acinetobacters (66.7%) were found to be positive for the carbapenemase production by MHT.

**Fig. 7:**
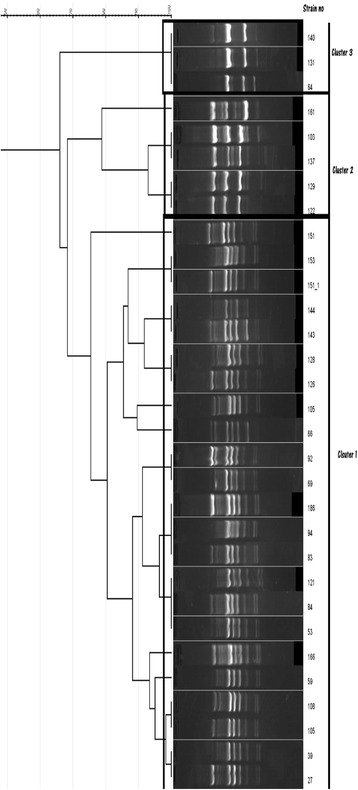
RAPD clusters of Acinetobacter isolates. *Isolate 105 included in the analysis twice as it was used as the internal control

The UPMGA based clustering of the RAPD results of *Acinetobacter* isolates with a tolerance of 4 revealed 3 clusters at 75% similarity. The predominant cluster (Cluster 1) consisted of 22 isolates (73.3% of all *Acinetobacter* isolates) with 21 of them clustering together even at 80% similarity. The second cluster (Cluster 2) consisted of 5 isolates (16.7% of all *Acinetobacter* isolates) and the third cluster (Cluster 3) consisted of 3 isolates (10% of all *Acinetobacter* isolates) (Fig. 7).

The RAPD clusters differed in sensitivity to some antibiotics and the cluster specific sensitivity patterns for these antibiotics are given in Table 2.

**Table 2:** Selected antimicrobial sensitivity testing results and MHT results of the RAPD clusters

Characteristic	Cluster 1 *n* = 22	Cluster 2 *n* = 5	Cluster 3 *n* = 3	*p* value**
Amikacin non-susceptibility^a^	17 (77.3%)	4 (80%)	1 (33.3%)	0.313
Gentamicin non-susceptibility	22 (100%)	3 (60%)	2 (66.7%)	0.013
Ceftazidime non-susceptibility	22 (100%)	5 (100%)	1 (33.3%)	0.007
MHT positive	14 (63.6%)	4 (80%)	2 (66.7%)	1.000

^a^Non-susceptible = intermediate sensitive + resistant

**p value calculated with the Freeman-Halton extension of Fisher’s exact test using Soper, D.S. (2016). Fisher’s Exact Test Calculator for a 2 × 3 Contingency Table [Software]. Available from http://www.danielsoper.com/statcalc

**Fig. 8:**
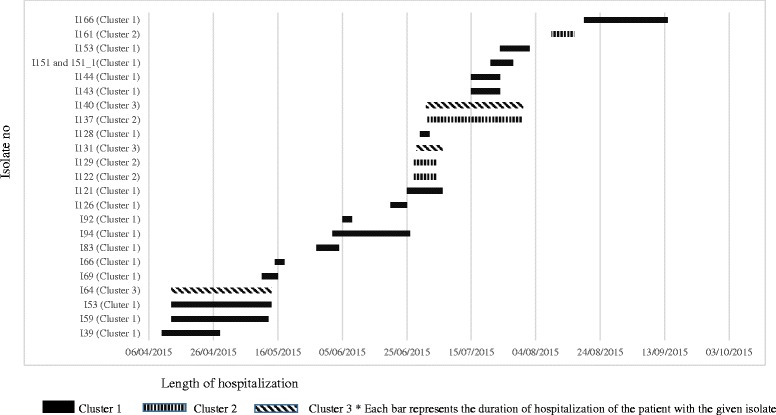
Timeline of ICU stay in patients with *Acinetobacter* spp

Analysis of length of stay in the ICU in patients in whom data were available (*n* = 24) indicated epidemiological links in the form of overlap in stay among many patients Fig. 8).

## Discussion

This study identified 25 coliforms (35.7%) out of 70 Gram negative isolates. All seven *E.coli* and all 14 *K.pneumoniae* isolates were found to be ESBL producers. A study on blood stream infections in Sri Lanka had identified that 22.5% of adult septic patients had ESBL producing organisms as causative agents [10]. Similarly, in a study conducted on urinary isolates, 40.2% had been identified as ESBL producing enterobacteriaceae [19]. Both these studies would have included more patients with community onset disease than the cohort of the current study, accounting for the differences in resistance rates. Considering mainly health care associated infections, a recent study performed in an Indian tertiary hospital founded that among 223 isolates of *K. pneumoniae,* 114 (51.1%) were ESBL producers [20]. A long term study at an Indian institute had identified ESBL prevalence to be as high as 80.9% within certain ICUs [21].

All *E.coli* isolates were found to harbour *bla*_CTX-M_. CTX-M has emerged to be the most common genetic determinant for ESBL production globally, including Sri Lanka [19, 22]. The most common genetic determinant of ESBL production among the *K.pneumoniae* isolates was *bla*SHV, followed by *bla*CTX-M. A separate study in Sri Lanka has also found a higher presence of *bla*SHV among *K.pneumoniae* isolates than *E.coli* isolates [19]. Similar findings have been reported from elsewhere in the world too [23]. A number of *K.pneunoniae* isolates (50%) were found to carry all three types of ESBL associated genes. It is alarming to notice this trend, particularly as the genetic determinants of resistance may be found on transmissible genetic elements.

The available therapeutic options for the treatment of ESBL-associated infections are limited due to co-resistance to various groups of antibiotics. Carbapenems are the the drug of choice against ESBL producers. However, with the increasing rates of ESBL producers, carbapenems are being used increasingly as empirical therapy. This in-turn drives the development of resistance to carbapenems. In this study six *Klebsiella pneumoniae* isolates (43%) were found to be non-susceptible for carbapenems and were confirmed to be carbapenemase producers by the MHT. A Sri Lankan study conducted from January to April 2012 had identified a carbapenamase production rates of 7.9% and 0% at two institutes in the Western province of Sri Lanka [6]. However, this study included isolates other than those obtained from ICUs.

In our study, which was conducted at a center over a period of 6 months, the majority of respiratory tract isolates were *Acinetobacter* spp. (*n* = 30, 43%). In a study conducted in 15 ICUs across Vietnam, the most frequently identified hospital associated infection related isolates were *A. baumannii* (*n* = 726, 24.4%) [24]. In a recent study conducted in Sri Lanka, out of 200 patients with VAP, 33.5% of the patient samples yielded *Acinetobacter* species [7]. Our study included organisms isolated from patients, irrespective of whether the patients had underlying Ventilator Associated Pneumonia (VAP) or not and some of the isolate could have been colonizers. However, if a patient develops subsequent VAP, it is likely that the colonizing flora would give rise to the infections; therefore, our sample selection could be justified coupled with the difficulty in establishing the diagnosis of VAP [25]. Furthermore, a recent long term study in the United States has identified that the presence of carbapenem resistant *A. baumannii* on surveillance cultures was strongly associated with the later development of infections with the same organisms, further validating our selection of samples [26].

In our study, all seven of the *E. coli* isolates, all 14 *Klebsiella pneumoniae* isolates and all 30 *Acinetobacter spp*. isolates could be defined as MDR as they were resistant to more than three antimicrobial classes. Out of the 15 *P. aeruginosa* isolates, only two MDR isolates (13%) were found to be MDRs. A cross sectional study which was conducted in Siriraj Hospital, Thailand considering all hospitalized patients who had positive culture for enterobacteriaceae, *P. aeruginosa* and *A. baumannii* during February to May 2012 revealed 48.8% of prevalence in overall MDR gram-negative bacteria. The percentage of MDR organism was 37.8% for ESBL producing enterobacteriaceae, 39.3% for carbapenem-resistant *P. aeruginosa* and 88.7% for MDR *A. baumannii* [27]. Our figures are somewhat higher than this study, which included patients from all units. The emergence of MDR Gram negatives in the ICUs is a major concern and needs urgent interventions in a country wide scale. As a number of genetic determinants of resistance can occur within the same mobile genetic elements (MGE), it would be interesting to identify the possible MGEs in our isolates.

The RAPD analysis of the four types of organisms studied revealed that in all types, a predominant cluster could be identified, rather than multiple clusters or singletons. This hints towards a possible within unit transfer of organisms rather than multiple acquisitions from outside environments. Similarly, graphical representations of the duration of hospitalization among the patients also demonstrate that there is overlap among the stay of patients carrying similar organisms belonging to given RAPD clusters as well as sporadic introduction of other cluster. Environment could be a potential source for the spread of the same cluster between patients who did not have an overlapping stay. These highlight the importance of adhering to proper infection control protocols within infection care units. Further analysis of isolates for a longer period of time would enable a time-series analysis of the different clusters.

While RAPD is a simple typing method, a typing method with more resolution might have given a better resolution that may have enabled a better differentiation of the strains. Performing a typing method such as MLST on selected RAPD clusters would strengthen the current results. However, the ability to perform RAPD in a relatively cost effective manner and the ability to analyze the fingerprint data using a free software that is easy to use with a graphical user interface, demonstrates that this method could be used in low cost settings. RAPD has also been integrated successfully in infection control programmes elsewhere [28].

There were several limitations in the current study. Our study was performed in a single center over a relatively shorter period. Therefore, time trends in resistance cannot be assessed. We also did not differentiate colonizing agents from agents causing infections. Genetic determinants of resistance was identified only among the enterobacteriaceae and only for ESBL production. Further studies need to look in to carbapenamase producers as well. The sensitivities were conducted using disc diffusion testing and minimum inhibitory concentrations (MICs) were not performed. Furthermore, we included isolates from respiratory specimen only. We also did not analyze the details in relation to the underlying conditions warranting ICU admissions. However, by using RAPD and hospitalization timelines, we could demonstrate the possible linkage of organisms included in the study.

## Conclusions

In conclusion, simple RAPD based typing followed by graphical analysis of hospitalization data can be used to identify trends in the presence and spread of resistance organisms in a cost effective manner. Data obtained in this manner could be a more convincing tool to persuade health-care staff to adhere to infection control protocols. Such surveillance could be made an integral part of the infection control programmes and could be used in possible outbreak situations to evaluate the spread and effectiveness of control measures.

## Abbreviations

ESBL: Extended spectrum β lactamases
HAI: Healthcare Associated Infections
ICU: Intensive Care Unit
MDR: Multi drug resistance
MHT: Modified Hodge test
RAPD: Random amplification of polymorphic DNA
